# A Machine Learning Approach for Movement Monitoring in Clustered Workplaces to Control COVID-19 Based on Geofencing and Fusion of Wi-Fi and Magnetic Field Metrics †

**DOI:** 10.3390/s22155643

**Published:** 2022-07-28

**Authors:** Ahmed M. Abd El-Haleem, Noor El-Deen M. Mohamed, Mostafa M. Abdelhakam, Mahmoud M. Elmesalawy

**Affiliations:** 1 Electronics and Communications Engineering Department, Faculty of Engineering, Helwan University, Cairo 11795, Egypt; ahmed_abdelkhaliq@h-eng.helwan.edu.eg (A.M.A.E.-H.); mabdelhakam@h-eng.helwan.edu.eg (M.M.A.); 2 Electrical and Communication Engineering Department, Faculty of Engineering, British University in Egypt (BUE), Cairo 11837, Egypt; 3 Computer and Systems Engineering Department, Faculty of Engineering, Helwan University, Cairo 11795, Egypt; nooreldeenmagdy@h-eng.helwan.edu.eg

**Keywords:** dynamic user-centric clustering, COVID-19, artificial intelligence, machine learning, deep learning, geofencing, digital signature, magnetic field

## Abstract

The ubiquitous existence of COVID-19 has required the management of congested areas such as workplaces. As a result, the use of a variety of inspiring tools to deal with the spread of COVID-19 has been required, including internet of things, artificial intelligence (AI), machine learning (ML), and geofencing technologies. In this work, an efficient approach based on the use of ML and geofencing technology is proposed to monitor and control the density of persons in workplaces during working hours. In particular, the workplace environment is divided into a number of geofences in which each person is associated with a set of geofences that make up their own cluster using a dynamic user-centric clustering scheme. Different metrics are used to generate a unique geofence digital signature (GDS) such as Wi-Fi basic service set identifier, Wi-Fi received signal strength indication, and magnetic field data, which can be collected using the person’s smartphone. Then, these metrics are utilized by different ML techniques to generate the GDS for each indoor geofence and each building geofence as well as to detect whether the person is in their cluster. In addition, a Layered-Architecture Geofence Division method is considered to reduce the processing overhead at the person’s smartphone. Our experimental results demonstrate that the proposed approach can perform well in a real workplace environment. The results show that the system accuracy is about 98.25% in indoor geofences and 76% in building geofences.

## 1. Introduction

The COVID-19 pandemic is causing turmoil around the world. Despite significant scientific advances in healthcare and technology, this pandemic remains a major threat. It can spread rapidly from one person to another, causing countless deaths. The World Health Organization (WHO) has announced COVID-19 a public health emergency [1] due to the challenges in controlling its spread. Many information technology advancements can be used to combat the epidemic, such as the Internet of Things (IoT), artificial intelligence (AI), machine learning (ML), and geofencing technologies [2]. In particular, IoT can help to mitigate the spreading of the virus by facilitating the process of monitoring and realization of COVID-19 control measures. IoT can be used to provide identification of persons and to collect different types of data required for managing the COVID-19 control measures. Machine learning can be used in various applications that can help to deal with COVID-19. Furthermore, geofencing technology can be used to control and monitor persons moving in/out of virtual boundaries to enforce COVID-19 control measures.

Researchers have applied machine learning techniques in different fields such as logistics [3,4], tourism [5,6], finance [7], and so on. In addition, machine learning and deep learning techniques can be adopted to provide effective solutions for the current problem of geofencing. In this approach, the workplace is divided into a set of geofences. Then, based on the nature and requirement of the work, the workplace environment will be clustered using the idea of a dynamic user-centric clustering [8]. Different wireless parameters and metrics play important roles as location signatures to apply such geofencing techniques, especially with applying machine learning with it. These metrics include the Wi-Fi basic service set identifier (BSSID) and the Wi-Fi received signal strength indication (RSSI) of the Wi-Fi access points (APs) and other smartphone sensors such as magnetometers and accelerometers.

In the literature, many recent works have been conducted to monitor and detect violations of defined COVID-19 control measures using information technology advancements. In [9], the authors introduce the “LakshmanRekha” smartphone app for home quarantine based on continuous user biometric authentication (CUBA), where biometric camera data and the global positioning system (GPS) location are collected from the smartphone sensors. This app consists of two modules, the front-end apps that consist of two apps, namely the administration app and patient app, and the home quarantined management (HQM) back-end. The authors in [10] present an IoT-Q-Band solution that can be used to prevent the spread of COVID-19. In this solution, a lightweight bracelet is worn by a quarantined person and connected to a smartphone application through Bluetooth technology. The person is registered by their personal details and the GPS parameters of their location as well as defining the geofence of this person. Convolutional Neural Network (CNN) is used in [11] to design a system for monitoring the violation of a home quarantine. This system consists of a software application, cloud database and storage, IoT node, and magnetic switch.

Signature Home is proposed in [12], an automated IoT-based geofencing technique to monitor the home quarantined persons, which exploits the special characteristics of networks, such as Wi-Fi and cellular networks, to decide the in/out status of the persons. In [13], the authors proposed a methodology to track COVID-19 zones, to enhance and tighten security rules. They monitor whether a person is entering or leaving a containment area and send an alert message to the person’s smartphone. A Geo-COVID approach is proposed in [14] based on a geofencing framework to manage and monitor the people’s movement using GPS location. In [15], a geofencing protection system is proposed for home quarantine and monitoring objectives, where GPS and GSM modules are used with a microcontroller-based system to be used as a bracelet. IoT and blockchain technology are used in [16] to collect and analyze the physiological and geographical data for quarantined people. This proposed system provides not only a real-time tracking for quarantined people but also protects their privacy and security.

An Android application is developed in [17] to inform persons within the containment zones to avoid entering these areas. This application updates the areas on a Google map to be defined as a containment zone and informs users if they enter these areas and also stores the international mobile equipment identity (IMEI) numbers of these persons to an online database. In [18], the authors propose a detection, tracking and alerting mobile application to manage the COVID-19 spread based on geofencing and ML technologies. This application can detect the presence of virus inside the persons through a ML model and provides an efficient tracking system to track the contacted persons. Then, an alerting system informs these persons with this state. However, the existing approaches for home quarantine [9,10,14,15,18] depend on the GPS parameters that do not work well and cannot be applied in indoor environments [19]. Furthermore, the offered solutions [11,15,16] are high-cost systems and require additional infrastructure.

Some of the other areas where IoT, AI, and geofencing technologies can find a way to prevent an epidemic are workplaces and the campus environment. Correct deployment of these technologies can help beat the spread of the virus in these areas. In this paper, an efficient approach is proposed for the workplace environment to control the density of the persons during working hours. This approach will split the workplace environment into a number of geofences, where each room is considered as a geofence. Then, the workplace environment is divided into different virtual cluster areas using a dynamic user-centric clustering scheme, where each person is associated with a set of geofences, forming its own cluster.

The proposed approach consists of three modules: a bracelet that will be paired with each person’s smartphone using Bluetooth technology, a mobile client app, and a web management platform that helps the workplace manager with applying different control measures. These control measures monitor the movement of persons in the workplace environment, limiting the capacity of the persons in all geofences, detecting whether persons are in the associated geofences, providing the total amount of time during which the person is detected outside their cluster, and alerting and alarming services when persons violate control measures. Different ML techniques are used to generate a unique geofence digital signature (GDS) for each geofence and each building in the workplace environment. Binary classification is applied to predict whether the person is in their cluster or not. The ML models are trained using a dataset of different metrics such as Wi-Fi BSSID, Wi-Fi RSSI and magnetic field data that can be collected with a smartphone without any requirement for additional hardware installation for all the pre-defined geofences. The proposed approach also attempts to reduce the processing overhead at the person’s smartphone.

The proposed approach also introduces the use of statistical features with magnetic field data for expanding its dimensionality and used the smartphone orientation features along with it. Different ML techniques are tested to find the most suitable one for the practical implementation and testing in real-life workplaces geofences.

It is worth mentioning that the focus of our approach is to be light enough to be effectively deployed on a large scale with low training time and scalable to different types of standard users’ devices and environments. Finally, experimental results are conducted in a real workplace with multiple chosen geofences. The results with the ANN deployed model showed that the system accuracy is about 98.25% in indoor geofences and 76% in building geofences.

The rest of the paper is organized as follows. Section 2 introduces the system model, the proposed clustering approach, and the description of the problem. The metrics used for generating the GDS are presented in Section 3. Section 4 provides different AI-based models that are used for generating a unique GDS for each geofence and doing a binary classification task. The performance results are discussed in Section 5. We conclude this paper in Section 6.

## 2. System Model and Problem Description

To monitor the control measures in the workplace environments, we propose an efficient approach to control the density of the persons during working hours. This approach can be used not only to control COVID-19 infection but also to monitor the employees in the workplaces. The proposed approach is based on geofencing and AI technologies. This approach will split the workplace environment into a number of geofences, where each room is considered as a geofence. According to the nature of the work, persons may want to move between different geofences. Therefore, the workplace environment is divided into different virtual cluster areas, where each person is associated with a set of geofences forming its own cluster. These clusters can be defined by the workplace manager to control the density of the persons during working hours.

### 2.1. System Model and Clustering Approach

In order to cluster the workplace environment, two types of clustering can be performed, which are the disjoint clustering and the user-centric clustering. In the former, persons are divided into groups, and the workplace is split into non-overlapping cluster areas, where each group of persons is assigned to a cluster area and persons in each cluster area cannot be in another cluster. However, they can be in any geofence in the same cluster area. In the latter clustering approach, each person is assigned to a cluster area that contains a group of geofences, and cluster areas of different persons can be overlapped together.

**Figure 1:**
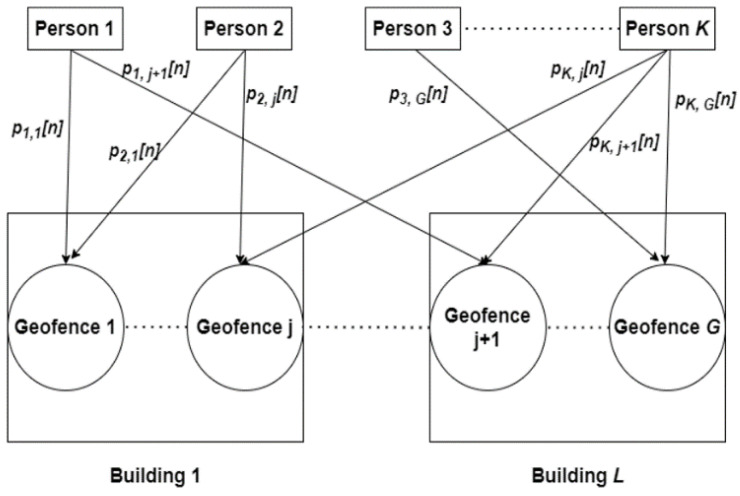
Dynamic user-centric clustering approach for the workplace environment.

Each clustering approach can be implemented in either a static or dynamic manner. In the static clustering approach, clusters are fixed, are defined prior and they change only when the manager of the workplace changes them manually by updating the control measures. Meanwhile, in the dynamic clustering approach, clusters are changed when the persons density changes and are based on the pre-defined control measures. According to the nature of the work, persons may want to move between different geofences in different cluster areas. Therefore, the user-centric clustering is the proper approach for clustering the workplace environment. Moreover, the dynamic clustering approach is more flexible than the static approach when the density of the persons changes during working hours. When the density of persons is high, each person should not move to any geofence that they are not associated with, i.e., not included in the cluster of this person. On the other hand, when the density is low, the associated geofences for each person can be increased. The proposed clustering approach for the workplace environment is illustrated in Figure 1.

Assume that the persons are split into three classes. The first class is a fully authorized person such as the manager of the workplace that can visit all the geofences. The second class is a partially authorized person that can join a number of associated geofences. Meanwhile, the third class is a unity authorized person that can join only one associated geofence. On the other hand, the geofences are split into two classes, i.e., public and private geofences. The public geofences are temporary associated areas such as break areas and bathrooms, where all persons have these geofences in their clusters. To control the capacity in the public geofences, some geofences are scheduled for specific times and specific durations such as break areas. Meanwhile, some geofences are scheduled for specific durations such as bathrooms. Meanwhile, the private geofences are permanently assigned to some persons according to the work nature. Denote K={1, 2, …, K} and G={1, 2, …, G} as a set of persons and geofences, respectively. It is clear that the fully authorized persons have all geofences in G in their own clusters and the partially authorized persons have a set of geofences P⊂G as associated geofences for them.

Let T represent the time duration of the workday. Suppose that T is split into N time intervals such that the duration of each interval n∈N={1, 2, …, N} is defined by $T_{n}$. To define the proposed dynamic user-centric clustering approach, let $\beta_{kg}\left[n\right]$ represent a binary indicator to define whether the person k has geofence g in its cluster at the time interval n, where $\beta_{kg}\left[n\right]$ can be given by

(1) $$\beta_{kg}\left[n\right]=\{\begin{array}{ll} 1, & \mathrm{if}\;\mathrm{the}\;\mathrm{person}\;k\;\mathrm{is}\;\mathrm{attached}\;\mathrm{with}\;\mathrm{the}\\ & \mathrm{geofence}\;g\;\mathrm{at}\;\mathrm{the}\;\mathrm{time}\;\mathrm{interval}\;n,\\ 0, & \mathrm{otherwise}. \end{array}$$

Let $G_{k}\left[n\right]$ denote the number of associated geofences for the person k at the time interval n, where the set of these geofences is represented by $G_{k}\left[n\right]=\left\{1,\;2,\;\ldots ,\;G_{k}\left[n\right]\right\}$. It is clear that $G_{k}\left[n\right]\subseteq G$ based on the class of the person k. In addition, the number of persons associated with any geofence g at the time interval n is denoted as $K_{g}\left[n\right]$, where the set of these persons is defined as $K_{g}\left[n\right]=\left\{1,\;2,\;\ldots ,\;K_{g}\left[n\right]\right\}$. Consequently, the proposed user-centric clustering approach is subject to the following requirements,

(2) $$\beta_{kg}\left[n\right]=1,\forall k\in K,\forall g\in G_{k}\left[n\right],\forall n\in N,$$

(3) $$\beta_{kg}\left[n\right]\in \left\{0,1\right\},\forall k\in K,\forall g\in G,\forall n\in N,$$

(4) $$\underset{g\in G_{k}\left[n\right]}{\sum }\beta_{kg}\left[n\right]=G_{k}\left[n\right],\forall k\in K,\forall n\in N,$$

(5) $$1\leq \underset{g\in G_{k}\left[n\right]}{\sum }\beta_{kg}\left[n\right]\leq G,\forall k\in K,\forall n\in N,$$

(6) $$\underset{k\in K_{g}\left[n\right]}{\sum }\beta_{kg}\left[n\right]=K_{g}\left[n\right],\forall g\in G,\forall n\in N,$$

where Equation (2) illustrates that the binary indicator $\beta_{kg}\left[n\right],$ is equal to one for all geofences in the defined cluster for the person k at the time interval n. Meanwhile, Equation (3) shows that the indicators $\beta_{kg}\left[n\right],\forall k\in K,\forall g\in G,\forall n\in N,$ should have binary values. Equation (4) indicates that the number of associated geofences in the cluster of the person k at the time interval n is $G_{k}\left[n\right]$, and the inequality in Equation (5) shows the limits on the number of associated geofences in the defined cluster for each person k at each time interval n based on the authorization classes of persons. In addition, Equation (6) indicates that the number of persons associated with the geofence g at the time interval n is $K_{g}\left[n\right]$. The manager of the workplace can control the density of the persons during working hours by setting the values $K_{g}\left[n\right],\forall g\in G,\forall n\in N$.

Define $p_{kg}\left[n\right]$ to represent the geofence presence probability (GPP) for each person k at the time interval n. The GPP defines the probability that the person k coexists inside the geofence g at the time interval n. Clearly, GPP can be changed during the working hours and can be defined by the manager of the workplace. Then, the probability $p_{kg}\left[n\right]$ can be defined for each time interval n as follows,

(7) $$0\leq p_{kg}\left[n\right]\leq 1,\forall k\in K,\forall g\in G,\forall n\in N,\;$$

(8) $$\underset{g\in G}{\sum }p_{kg}\left[n\right]=1,\forall k\in K,\forall n\in N,$$

where Equation (7) indicates that the probability $p_{kg}\left[n\right]$ is a positive value between 0 and 1. Meanwhile, Equation (8) illustrates that the sum of GPP of all geofences for each user k at each time interval n is equal to one.

### 2.2. Problem Description

Each person in the workplace environment would wear a bracelet that will be paired with their smartphone using Bluetooth technology to ensure that the person carries the smartphone during moving. This can be useful to notify the workplace manager when the person takes off the bracelet and the connection of the Bluetooth is terminated during the working hours. Different metrics are collected using the smartphone of the workplace manager in all the pre-defined geofences and are used for generating the unique GDS for each geofence. In addition, the manager of the workplace will generate a GDS for each building in the workplace environment.

During working hours, the smartphones of the employees will collect the pre-defined metrics periodically at time intervals of $T^{p}$ when the smartphone’s accelerometer senses that the person is moving. This can help to reduce the processing overhead on the person’s smartphone. After that, the collected metrics are used to generate the GDS for the geofence that the person is located in. Then, it will compare with the GDSs of their associated geofences that are defined in their own cluster to identify whether the person is in their cluster or not.

**Figure 2:**
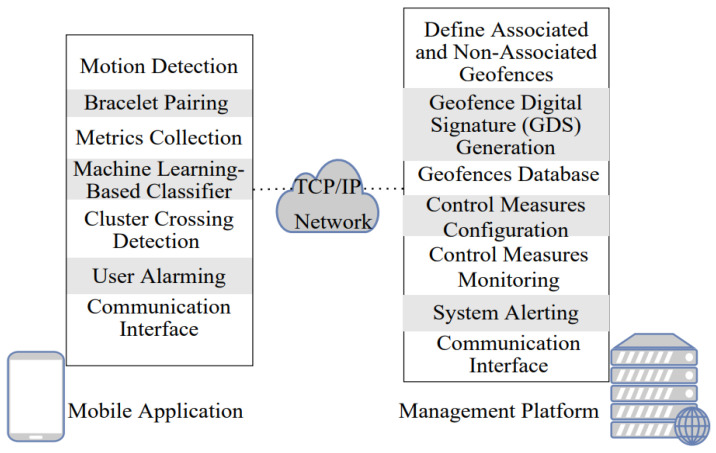
Functional block diagram for the proposed approach.

Layered-Architecture Geofence Division (LAGD) method is considered in this paper. First, the generated GDS is compared with buildings’ GDSs to identify whether the person is inside or outside the buildings. When there is a matching between the generated GDS and the GDS of any building, sort the set ${ℱ}_{k}\left[n\right]=\left\{1,\;2,\;\ldots ,F_{k}\right\}$ that contains the associated geofences for the person k at the time interval n that located inside this building in descending order based on the GPP. After that, the generated GDS is compared with the GDS of the current building and the GDS of all the associated geofences in ${ℱ}_{k}\left[n\right]$ based on the sorted list considering starting with the GDS of the current building. In the case of mismatching, wait for a fixed time margin $T^{s}$ and repeat the above steps. This can help to reduce the processing overhead at the employee’s smartphone. In the case of matching between the generated GDS and the GDS of any geofence in ${ℱ}_{k}\left[n\right]$, this indicates that the person is inside their own cluster of associated geofences. If the GDSs are mismatched during a pre-defined violation time interval $T^{v}$, an alert will be generated to the manager of the workplace to inform them of this violation to take proper action. The functional block diagram for the proposed approach is illustrated in Figure 2.

## 3. Metrics for Geofence Digital Signature (GDS) Generation

### 3.1. Metrics for Signature Generation

The system idea needed to be robust and ideal for any environment without the effort to make any sort of infrastructure. Different metrics that can be accessed from any traditional commercial smartphone were studied and collected. Various smartphone sensor-based metrics were used to indicate the IO state as in [20], but these sensor-based metrics such as the light sensor and GPS have many disadvantages. The light sensor lacks the generalization on the same environment, the GPS is not an optimal indication for indoor environments with high battery consumption and both provides a low accuracy of detection in general [21]. Then, we decided to work on the metrics that would be specific to each workplace, will not change with time and have the optimal battery consumption.

In this subsection, different metrics are studied for generating the digital signature for any geofence in the workplace environment. First, Wi-Fi wireless metrics such as Wi-Fi basic service set identifier and Wi-Fi received signal strength indication parameters are used for GDS generation. This is due to the availability of them, as they can be collected in smartphones without any requirement for additional hardware installation. Wi-Fi BSSID can be considered as a distinctive identifier for each Wi-Fi AP [22] that characterizes the medium access control (MAC) address for the Wi-Fi AP. However, Wi-Fi BSSID can be collected even if the person is far from the AP with an appropriate distance. Therefore, Wi-Fi RSSI parameters are essential to be used along with the Wi-Fi BSSID to indicate the distance from the Wi-Fi AP for any person. The used Wi-Fi wireless metrics of all neighboring Wi-Fi APs can be collected by scanning the smartphone’s Wi-Fi to be used in digital signature generation for any geofence in the workplaces.

To increase the GDS robustness, the magnetic field can be used along with the Wi-Fi wireless metrics to uniquely identify each geofence and detect the crossing of its virtual borders. The magnetic field can be sensed by the magnetometer equipped in smartphones. The magnets affect the Earth’s magnetic field and construct a unique signature that can be detected by the smartphone’s magnetometer and used to track the geofences violation. The magnetic field of the magnets depends on the magnetic moment of the magnet, relative position to the smartphone’s magnetometer, and the magnetic permeability. Therefore, the three-axis parameters of the magnetic field $S_{x}$, $S_{y}$ and $S_{z}$ sensed by the magnetometer can be given by [23].

(9) $$\left(\begin{array}{c} S_{x}\\ S_{y}\\ S_{z} \end{array}\right)=\frac{{µ}_{0}}{4\pi r^{5}}\left|\begin{array}{ccc} 3x^{2}-r^{2} & 3xy & 3xz\\ 3yx & 3y^{2}-r^{2} & 3yz\\ 3zx & 3zy & 3z^{2}-r^{2} \end{array}\right|\left(\begin{array}{c} M_{x}\\ M_{y}\\ M_{z} \end{array}\right),$$

where x, y and z are the distance between the magnet and the magnetometer in the *x*-axis, *y*-axis, and *z*-axis, respectively. Meanwhile, r denotes the norm distance from the magnet to the smartphone’s magnetometer and ${µ}_{0}$ represents the magnetic permeability. Moreover, $M_{x}$, $M_{y}$ and $M_{z}$ denote the magnetic moment of the magnet.

### 3.2. Spatial Features for Magnetic Field

The smartphone’s magnetometer can collect the three-axis parameters of the magnetic field $S_{x}$, $S_{y}$ and $S_{z}$. In addition, the pitch P, roll R, and azimuth ψ parameters can be sensed by the smartphone’s accelerometer. These last three parameters are the output from the mobile orientation sensors or the position sensors which are used in determining the smartphone’s orientation [24]. The magnetometer field strength S can be given by

(10) $$S=\sqrt{S_{x}^{2}+S_{y}^{2}+S_{z}^{2}}.$$

According to [25], the horizontal and vertical parameters of the magnetic field $S_{H}$ and $S_{V}$ can be calculated as follows

(11) $$S_{V}=-S_{x}\sin\left(P\right)+S_{y}\sin\left(R\right)+S_{z}\cos\left(P\right)\cos\left(R\right),$$

(12) $$S_{H}=\sqrt{S^{2}-S_{V}^{2}}.$$

Using the parameters $S_{H}$ and $S_{V}$ in the GDS generation can help in reducing the magnetometer orientation effect. Therefore, the nind features $S_{x}$, $S_{y}$, $S_{z}$, P, R, ψ, S, $S_{V}$, $S_{H}$ are used to represent the spatial features for the magnetic field. Each feature is represented by the mean and the interquartile range (IQR) statistics, which can be given as follows,

(13) $$\mu =\frac{1}{c}\overset{c}{\underset{i=1}{\sum }}x_{i},$$

(14) $$IQR=Q_{3}-Q_{1},$$

where μ is a central value of the data. Meanwhile, IQR is the difference between the upper quartile $Q_{3}$ corresponds with the 75th percentile of the data and the lower quartile $Q_{1}$ corresponds with the 25th percentile of the data.

## 4. ML-Based Models for GDS Generation and Matching

In this section, we discuss the proposed ML-based techniques for generating the GDS for our geofenced workplaces. The idea of applying this ML approach is for performing a binary classification task to identify the inside/outside (IO) status of a user to determine whether the user is inside their pre-defined cluster of geofences. This is applied by fetching the user’s current geofence metrics by a developed mobile application. The output or the prediction of the ML model would be the indication of the presence of the users inside is associated cluster or not. If not, an alert would be sent to the admins of the web management system for taking the proper action. As we would be exposed to more than one geofence for each user, we still used the binary classification approach instead of a multi-class one. This is done to have the ability to add more geofences later as separate models to the system, creating new clusters or modifying them.

### 4.1. Features Collection and Extraction

The features collection would be the first step for building our ML models for the GDS generation. The chosen metrics were the Wi-Fi BSSID and Wi-Fi RSSI data along with the magnetometer different metrics Sx, Sy, Sz, S, $S_{H}$, $S_{V}$, P, R and ψ. So, after some tests, we decided to go with the first top-five BSSIDs and RSSIs from the Wi-Fi metrics which are scanned by the smartphone and those would make our first ten features in our dataset. Then, they would be followed by the nine features of the magnetometer and accelerometer sensors. The last thing would be our label which indicated the IO status in a certain geofence dataset.

The availability of the Wi-Fi metrics inside different workplaces and the changing nature of the magnetic field inside of buildings from the outdoor environment make them ideally good for our tests. Furthermore, the addition of the orientation parameters of the user’s smartphone supported the ML model accuracy as an indication of the real movement and placement of the smartphone in the workplace environment. This was done during the collection of the dataset features, as we tested the user’s smartphone usage by putting it on tables, holding it to browse some feed and messages, and walking with it in the trouser pocket.

The datasets for each testing location were collected by a developed Android application on Android Studio Arctic Fox (2020.3.1) edition running on a Xiaomi Redmi 9T. It is equipped with an Octa-core Qualcomm SM6115 Snapdragon 662 chip and Adreno 610 GPU with 4 GB random access memory (RAM) on Android 11 (API 30). The datasets were collected at 20 Hz (20 samples in 1 s) by the Android application in which the magnetometer and the accelerometer vary in each sample, while the Wi-Fi metrics are captured differently as we could get one new sample every 2.5–3 s from the Wi-Fi sensor.

These features were collected as a time-series data to make some statistical calculations on them. This approach was applied to increase the dimensionality of the magnetic data further for more features extraction. These datasets were in the range of 15,000 samples for each geofence dataset we tested on. This size of samples was acceptable in our tests. We could have increased the number of samples more, but due to the relatively small size of the geofences of different rooms or halls in real life, there is not much variety in the wireless metrics and magnetic data. The readings would be repeated, which causes a degradation in the performance of our ML techniques. On the other hand, less redundant data will lead to less possibility of making decisions based on redundant data/noise, which reduces any future overfitting.

Furthermore, the features are used interchangeably with different preprocessing steps according to the used model type in each state or situation, this is discussed later in Section 4.2 and Section 5.

### 4.2. TSMF Data Preprocessing

The datasets for the tested geofences are collected as time-series data at 20 Hz from the magnetometer and accelerometer sensors, and the Wi-Fi metrics were nearly at 0.3 Hz. Before going into details, it is worth mentioning that the reason for increasing and expanding our features from just the traditional magnetic field strength to having nine features of magnetic data was for achieving the possibility of using them on their own. Due to the low dimensionality of magnetic field strength alone, this is done mainly for covering the case of the absence of Wi-Fi signals in any location.

Different statistical operations were applied differently to our metrics in what is called the Time-Series Metrics Fusion (TSMF). There are three preprocessing TSMF situations in which the datasets of our system architecture are tested: magnetic data only; Wi-Fi metrics only; and Wi-Fi with magnetic data. At each time point we tested a different portion of the features.

First, the magnetic field and orientation TSMF. This is done by processing our 20 samples into one reading using two statistical operations, the Mean and Interquartile range. So, 20 samples of the nine features are taken each second and their mean is calculated to diminish the 20 samples into one single reading. The IQR is calculated in the same manner. Then, the nine means and nine IQRs are concatenated together to get 18 features with the addition of the IO indication label. These two operations were chosen specifically after some conducted tests between some other statistical operations such as variance, kurtosis, etc. The final preprocessing steps are done by splitting the data into 80% training dataset and the rest is used as a testing dataset. The traditional encoding of the IO state into 0 and 1 is as inside and outside labels, respectively. The last step before training the model is to standardize the data or use the StandardScaler, which standardizes the features by subtracting the mean and then scaling it to unit variance [26]. Unit variance means dividing all the values by the standard deviation and this method is applied to all our features of magnetic data.

Secondly, the Wi-Fi metrics TSMF. The features have the first five BSSIDs and their RSSIs which form 10 features, then the IO label is added to each sample. The number of samples were in the range of 600–900. As the RSSI varies from 0 to −100 dBm, the absence of the measurement of any of the top five Wi-Fi’s RSSI is marked as −100 dBm to make our model converge to higher results [27]. The corresponding missing BSSID of an RSSI would also get a value of −100 dBm to indicate its absence. During GDS matching, if an unseen Wi-Fi network BSSID is detected as a new input to the trained model, it is treated as the missing one with a value of −100 to prevent the confusion of the model and the tests showed the expected results. To prevent and overcome the problem of unseen Wi-Fi networks after training on certain data and the addition of new permanent Wi-Fi signals to a geofence location, a technique is applied by retraining the model at certain time points (e.g., during idle state, every night) using a new batch of collected wireless metrics so the model can identify them in future inferences. The last part is the encoding and labeling of the BSSIDs features in each row in the dataset, followed by the same steps of encoding the IO state, splitting the dataset, and the standardization of it.

Thirdly, the usage of the two types of features after the preprocessing steps were evaluated. We ended up with a total of 18 magnetic data features in addition to 10 Wi-Fi metrics features for a total of 28 features with the IO label.

### 4.3. ML-Based Classification Models

Different ML techniques [28] can be used to provide effective solutions for various real-life problems. Before studying the ML techniques, it is better to understand the difference between supervised and unsupervised categories of machine learning [29]. Unsupervised learning is when the model is trained with unlabeled data. This means that the model will have to find its own features and make predictions based on how it classifies the data. Supervised learning is when the machine learning model is trained using labeled data. This means that you have data that already have the right classification associated with them. One common use of supervised learning is to help you to predict and classify a class from a set of classes based on the input data. With supervised learning, the models would need to be rebuilt after acquiring new data to make sure that the predictions returned are still accurate. The ML-based supervised techniques that are used in our experiments are discussed in this section briefly.

#### 4.3.1. Support Vector Machine

**Figure 3:**
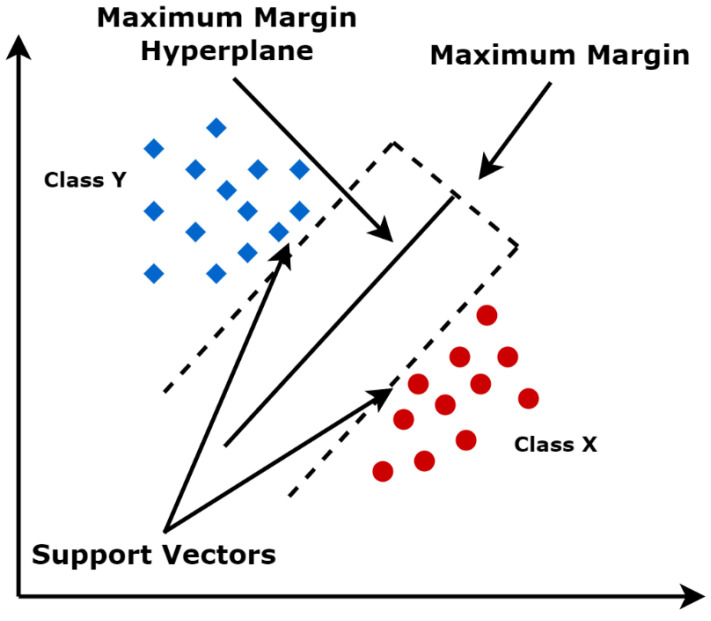
Support vector machine.

Support vector machine (SVM) is one of the most popular supervised learning algorithms [30]. SVM is used for classification and regression problems. However, primarily, it is used for classification problems in machine learning. The SVM algorithm’s main goal is to create the best line or decision boundary that can segregate n-dimensional space into classes to facilitate putting the new data point in the correct category in the future. This best decision boundary is called a hyperplane. Then, extreme cases are chosen that are called support vectors to help in creating the hyperplane. That is why it is called support vector machine. Figure 3 illustrates the idea in which there are two different categories that are classified using a decision boundary or hyperplane.

#### 4.3.2. k-Nearest Neighbor

**Figure 4:**
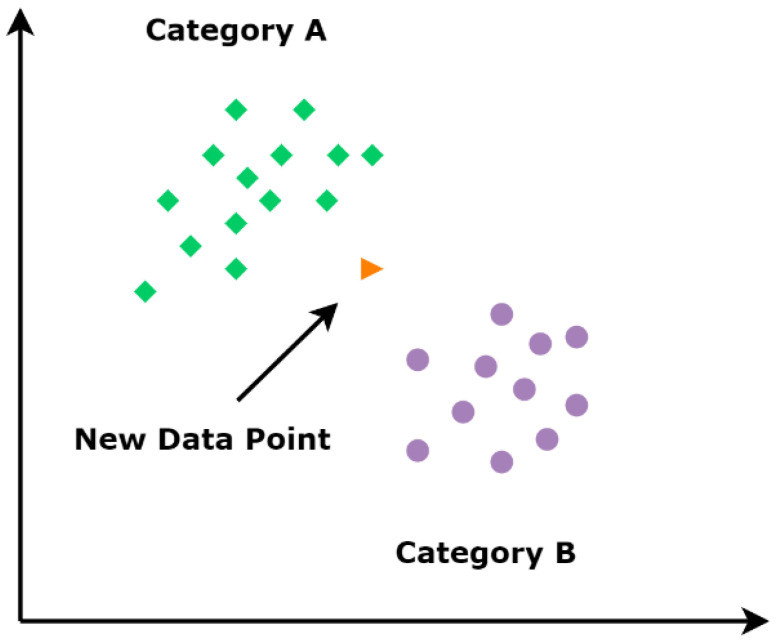
k-Nearest neighbor.

kNN is mostly used for classification problems [31]. kNN uses the similarity between the new data and available data categories to put the new data into the most similar available category. kNN algorithm at the training phase stores the dataset and when it gets the new data it classifies these data into a category that is like the new data. The kNN algorithm can be used for identification, as it works on a similarity measure. The kNN model will find similar features of the new data to the categorized data. Figure 4 illustrates the adding of a new data point in a cluster.

#### 4.3.3. Random Forest

**Figure 5:**
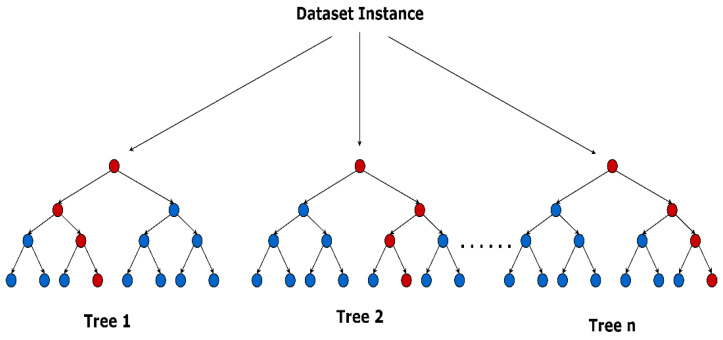
Random forest.

Random forest is a popular machine learning algorithm that belongs to the supervised learning technique. Random forest can be used for both classification and regression problems in ML [32]. It is a combination of tree classifiers where each tree is generated with a random vector from the input vector. It uses the idea of ensemble learning, which is a process of combining multiple classifiers to solve a complex problem and to improve the performance of the model. Random forest is a classifier that contains several decision trees on various subsets of the given dataset and takes the average to improve the predictive accuracy of the dataset. Instead of relying on one decision tree, the random forest takes the prediction from each tree, and based on the majority votes of predictions it predicts the final output. A greater number of trees in the forest leads to higher accuracy and prevents the problem of overfitting. Figure 5 shows the trees of a random forest, where the red circles represent the final class from each decision tree.

#### 4.3.4. Artificial Neural Network (ANN)

Artificial neural network is a term that refers to a biologically inspired sub-field of artificial intelligence modeled after the brain. An artificial neural network is based on biological neural networks that construct the structure of the human brain, and it is a computational network [33]. Like a human brain has neurons interconnected to each other, artificial neural networks also have neurons that are linked to each other in various layers of the networks. These neurons are known as nodes. The artificial neural network primarily consists of three layers: the input layer, which, as the name suggests, accepts inputs in several different formats; the hidden layer presents in-between input and output layers and performs all the calculations to find hidden features and patterns; and the output layer, which, as the input goes through a series of transformations using the hidden layer, finally results in output that is conveyed using this layer. The artificial neural network takes input $X_{i}$ and computes the weighted sum of the inputs and includes a bias b. This computation is represented as follows,

(15) $$\overset{n}{\underset{i=1}{\sum }}W_{i}X_{i}+b.$$

**Figure 6:**
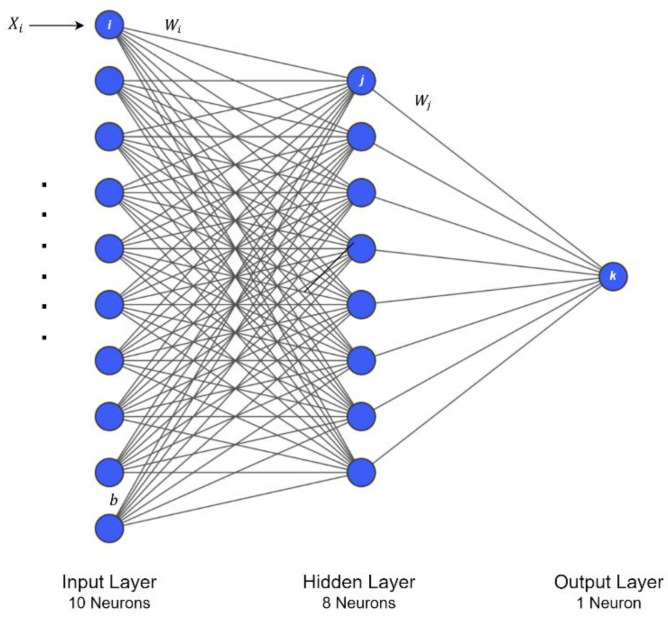
Artificial neural networks.

This equation is then passed to an activation function to produce the output. Activation functions choose whether a node should fire or not. Only those who are fired have an effect on the output layer. There are distinctive activation functions available that can be applied depending on the task we are performing. Figure 6 shows a neural network architecture.

#### 4.3.5. Convolutional Neural Network (CNN)

**Figure 7:**
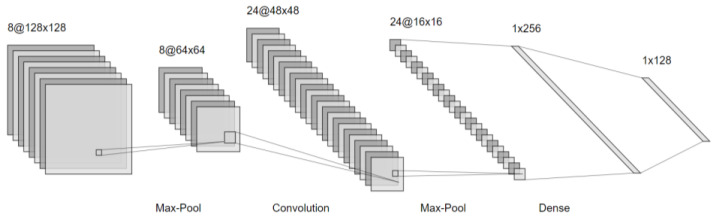
Convolution neural networks.

Another deep learning approach is tested which is the convolutional neural network (CNN). CNN is a form of deep neural networks that are specially designed and optimized for image classification [34]. CNN has been shown to deliver significantly higher classification accuracy as compared to conventional feed-forward ANNs due to their enhanced pattern recognition and feature extraction capabilities. In the context of a convolutional neural network, convolution is a linear operation that involves the multiplication of a set of weights with the input such as the traditional neural network. Given that the technique was designed for two-dimensional input, the multiplication is performed between an array of input data and a two-dimensional array of weights, called a filter or a kernel. The filter is smaller than the input data and the type of multiplication applied between a filter-sized patch of the input and the filter is a dot product. Using a filter smaller than the input is intentional as it allows the same filter (set of weights) to be multiplied by the input array multiple times at different points on the input. Specifically, the filter is applied systematically to each overlapping part or filter-sized patch of the input data, left to right, top to bottom. This systematic application of the same filter across an image is a powerful idea. If the filter is designed to detect a specific type of feature in the input, then the application of that filter systematically across the entire input image allows the filter an opportunity to discover that feature anywhere in the image. Figure 7 displays a CNN layers architecture.

### 4.4. Training and Testing Processes

After the training and testing process of the models on our geofences datasets, the suitable ML model parameters were discovered and are briefly discussed in this section. First are the classical machine learning models. In the SVM model, we have C equals 1.0, kernel: rbf, and gamma: scale. The kNN with n_neighbors is equal to 5. Then there is a random forest model with n_estimators equal to 100, max_depth: none, and max_features: auto.

Second are the deep learning models. The ANN model consists of three layers: input, hidden and output classifier dense layer with (10–8–1) neurons with 10 input features. Each layer has a ReLU activation function except the output with a sigmoid one recommended for binary classification. The optimization algorithm and the loss function are the Adam optimizer and the binary cross-entropy, respectively. The training epochs equal 150 with early stopping that is applied to prevent overfitting of the training process. In the CNN model, the difference in the preprocessing step changes from the StandardScaler to the MinMaxScaler [35]. For each value in a feature, the MinMaxScaler subtracts the minimum value in the feature and then divides it by the range. The range is the difference between the original maximum and the original minimum. MinMaxScaler preserves the shape of the original distribution. The required range for the feature returned by MinMaxScaler is 0 to 1. To make the data ready as an input to the CNN layers, we reshaped them from an array of 10 by 1 to an array of 5 × 2 × 1 in the case of Wi-Fi metrics, 9 × 2 × 1 in case of magnetics data and 14 × 2 × 1 in case of using all the features. The model has three layers of convolutional 2D layers with (64–128–256) filters, a kernel of 2 × 2 then 1 × 1 and the input size has dimensions 5 × 2 × 1 in the case of Wi-Fi metrics. We used Conv 2D instead of Conv 1D due to reshaping the data as a 2D image in the CNN models after using the TSMF approach on the time series data collected. This adjustment increased the accuracy by 2% to 3% using our dataset. The activation functions are ReLU. Due to the small input size, this CNN model does not have max-pooling layers. Finally, we will flatten the output of the CNN layers, feed it into two fully-connected dense layers with (100–50) neurons and then to a sigmoid layer for binary classification with training epochs also equal to 150 with early stopping applied. Both ANN and CNN had batch sizes equal to 32. Some other parameters used were the validation split with a value of 0.1 and model checkpoints.

During the implementation and testing, we used TensorFlow and Keras library [36] for deep neural networks and Scikit-learn [37] for the classical machine learning models implementation and the typical machine learning operations.

## 5. ML Setup and Results Analysis

### 5.1. ML Expermental Setup

**Figure 8:**
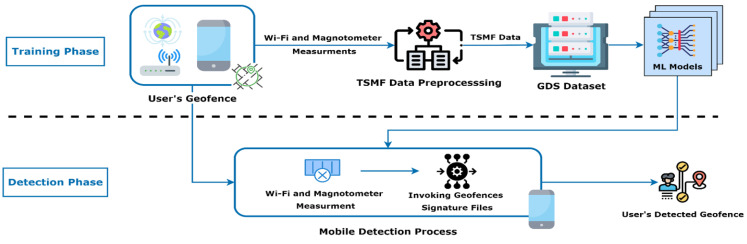
ML system operation diagram.

The operating idea of the system is illustrated in this section. The ML system operates in two modes, a training phase and a detection phase, as shown in Figure 8. The training phase involves the collection of the dataset of our chosen features in each of the pre-defined geofences for training the ML models by a smartphone admin application. Then, we apply the TSMF preprocessing to generate our required GDS datasets to train our chosen ML models. On the other hand, in the detection phase, each user’s associated geofences would have their own ML model deployed locally on their smartphone client application. This would detect the user’s location periodically at a defined time interval. So, we will detect if the user is inside their cluster or not. Each of the assigned clusters will be set to cover certain rooms or halls to manage the density of the people for monitoring the movement of employees in the workplaces. The real-time detected locations and the GDS of the users are uploaded to the web management system for tracking them automatically. Each group of users will be assigned by the system admin to a cluster of geofences based on the nature and the requirement of the work and the workplace environment.

**Figure 9:**
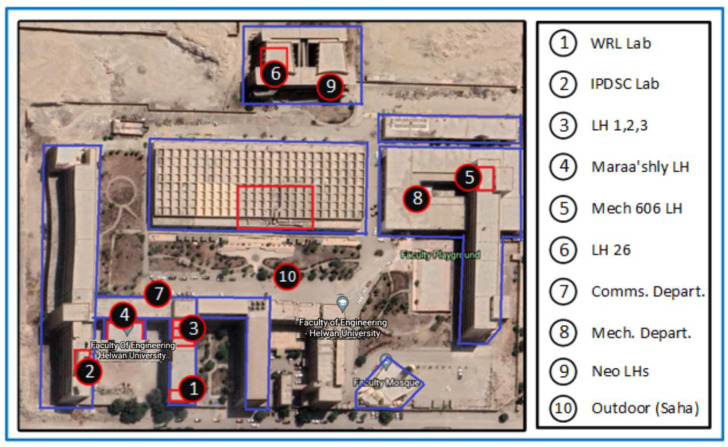
FE-HU top-view map.

**Figure 10:**
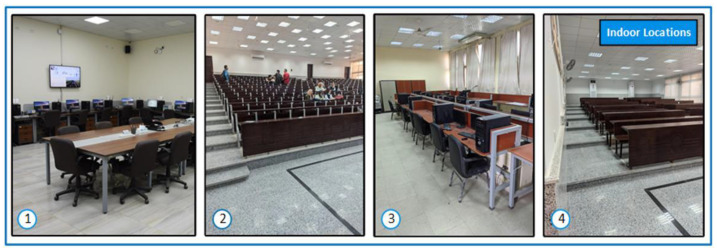
Images of indoor locations.

**Figure 11:**
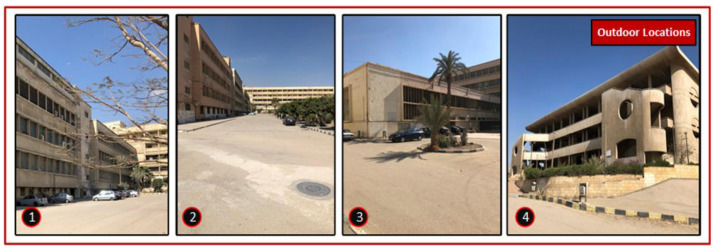
Images of outdoor/buildings locations.

The detection of the user’s location occurs by the ML prediction function on the mobile application. It is invoked periodically at regular time intervals to collect the wireless metrics and outputs a location. If the user is a student in a university, as an example, they will be assigned with the cluster of certain lecture halls 1, 2, and 3, where they take most of their lectures in hall 1. However, certainly, they are not assigned to the workers’ offices. The prediction function will have high priority weights for detecting their location with the model of lecture hall 1 than the other two. Therefore, the sequence of checking would be lecture hall 1, 2 then 3. If there is a matching, the generated output is the geofence name for any geofence in that user’s cluster. This implies that this person is inside their cluster, but if the output is outside each one of them after a predefined violation time interval, this indicates that the person is outside their pre-defined cluster. Then, an alarm will be produced to the user through the mobile application and an alert will be generated in the web management platform to inform the supervisors and authorized personnel of this violation to take proper action. The number of persons inside a certain geofence is calculated in the web management platform to monitor the density of people and for the implementation of our idea of dynamic clustering. The system admins set some pre-defined control measures so that the clusters change over time according to the density of individuals. For testing the system, we chose three different buildings with five lecture halls and two labs to represent the proposed buildings and indoor geofences in the Faculty of Engineering, Helwan University, Cairo, Egypt (FE-HU) as a workplace geofence to represent a real practical implementation of our system. The proposed approach was tested on three buildings: Communication Department, Mechanical Department, and the Neo Lecture Halls Building. Inside them, we chose five lecture halls: Lecture Hall 1, Lecture Hall 2, Maraa’shly Lecture Hall, Mech 606 Lecture Hall and Lecture Hall 26. Two other labs were chosen, the Wireless Research Lab (WRL) and Innovation and Product Development Support Center (IPDSC). Figure 9 shows the buildings and the chosen locations inside them. Figure 10 and Figure 11 have some images of the indoor and outdoor locations we tested in.

### 5.2. Tests and Results Analysis

Now the evaluation and the results of the developed ML models after training and testing can be discussed. The results analysis was carried out by applying Wi-Fi and MF models separately, and the combined WF-MF models on three different indoor locations and other three different buildings (Outdoor) locations. From now on, we call these different types of models WF-ML, MF-ML and WF-MF models. The target of this analysis is to find the best-used metrics in different situations.

**Figure 12:**
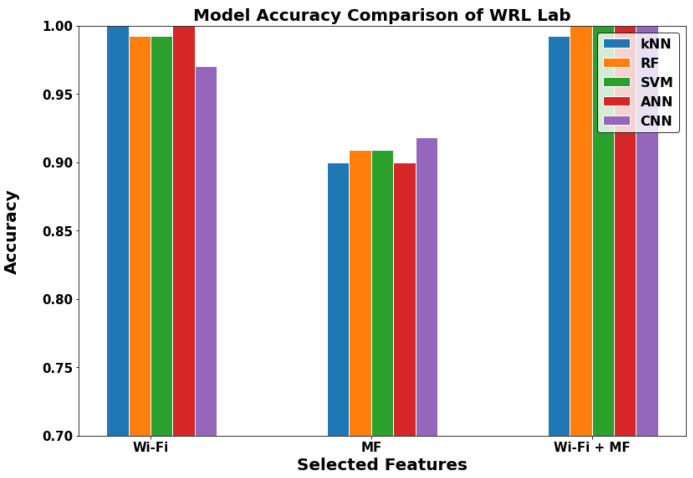
The models comparison in WRL Lab (Indoor).

**Figure 13:**
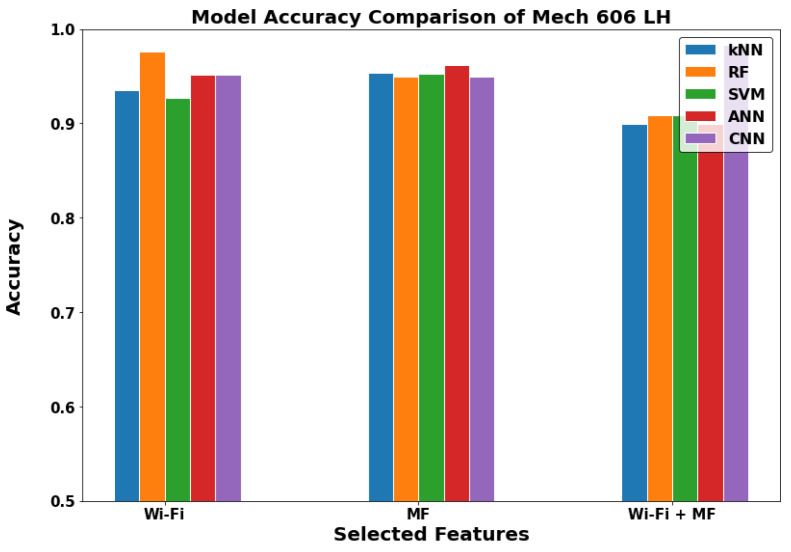
The models comparison in Mech 606 LH (Indoor).

**Figure 14:**
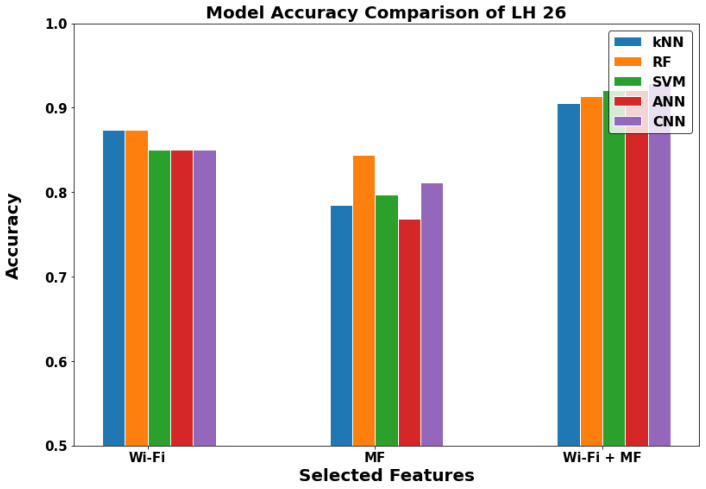
The models comparison in Lecture Hall 26 (Indoor).

In Figure 12, Figure 13 and Figure 14, we can see that the results of the indoor locations indicate different behavior. This is due to the nature of the datasets in each location and the availability of the metrics for the GDS generation. In the WRL Lab, the WF-ML and the WF-MF show nearly the same performance, and they are outperforming the MF-ML. In Mech 606 LH, WF-ML and MF-ML does not have a significant change, and WF-MF is worse than them. In LH 26, WF-MF has superior performance due to the low number of Wi-Fi access points in this LH. We can conclude by studying the three locations that the WF-ML is a better model for the indoor locations in the case of the availability of at least two or three Wi-Fi APs. WF-MF was good in two cases but having a model inference with all these parameters uses a large amount of processing power and time in the long range.

**Figure 15:**
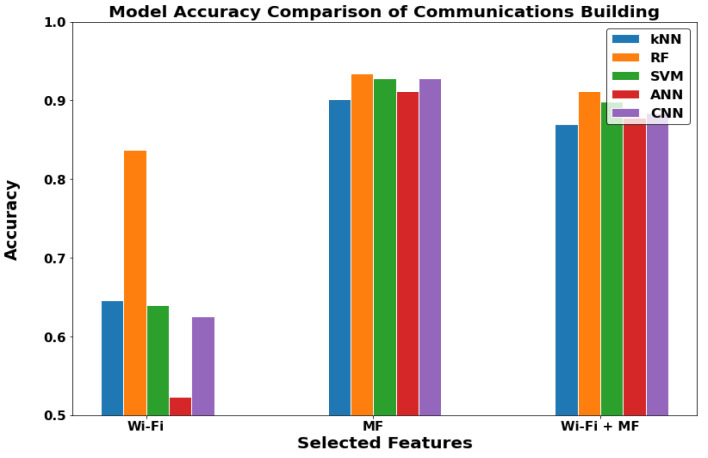
The models comparison in Communications department building (Outdoor).

**Figure 16:**
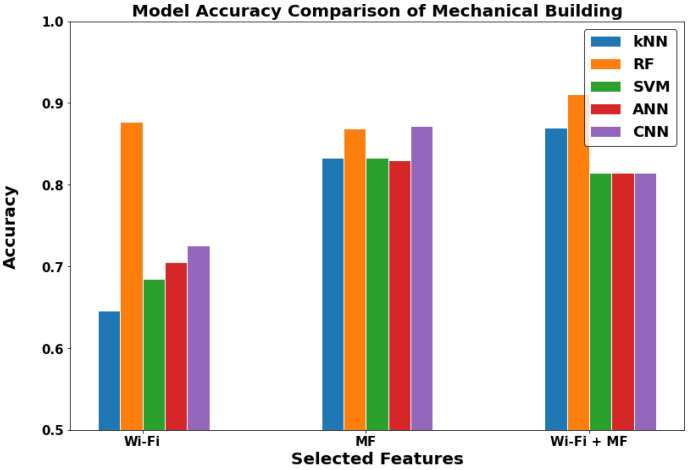
The models comparison in Mechanical department building (Outdoor).

**Figure 17:**
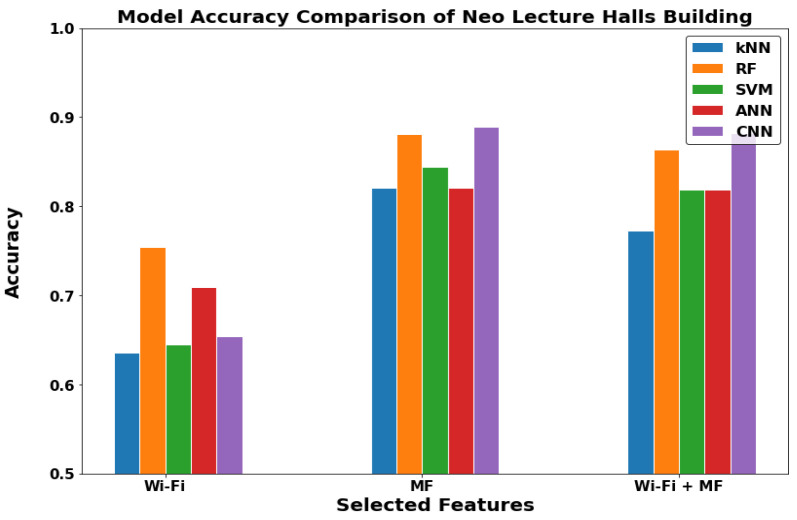
The models comparison in Neo Lecture Halls building (Outdoor).

The buildings or the outdoor environment layer in Figure 15, Figure 16 and Figure 17 can now be discussed. In the communications department building and Neo Lecture Halls building, the MF-ML have noticeably higher performance. The mechanical department building had better performance with WF-MF. It is important to note that most of the outdoor environments lack the presence of Wi-Fi coverage. Therefore, we did not get good results with WF-MF or WF-ML as in the first two locations discussed. Thus, we can conclude that MF-ML is the best model for the buildings (outdoor) locations.

**Figure 18:**
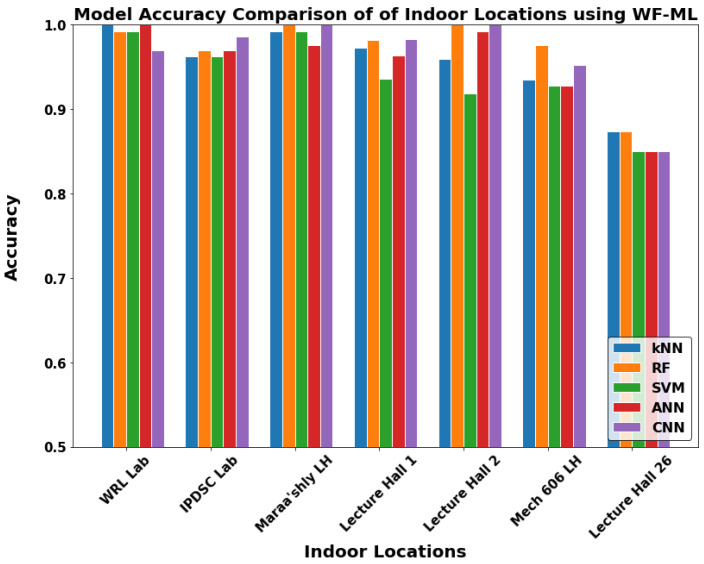
The models accuracy in all indoor geofences using WF-ML.

**Figure 19:**
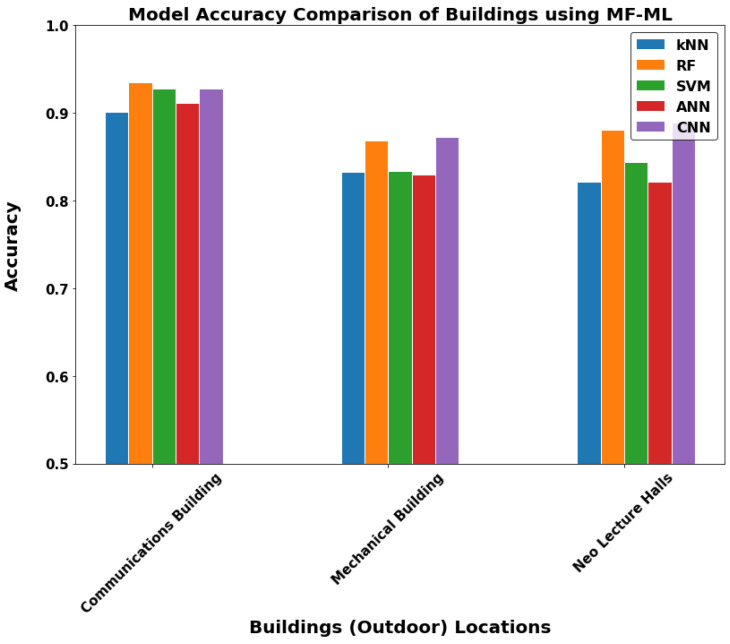
The models accuracy in all outdoor buildings geofences using MF-ML.

After we chose the WF-ML for the indoor locations layer and MF-ML for the buildings layer, Figure 18 and Figure 19 show the results of each location we tested on. The performance of the WF-ML models in Figure 18 is above the 95% accuracy taking into consideration that each indoor location offers a challenge to the ML models. Each location contains at least two or three Wi-Fi access points that are detected by the mobile application. We can see that the best models are the Random Forest, ANN, and CNN as each one has an advantage to a specific case. The random forest is mostly the highest accuracy model as the classical machine learning models do not require large datasets. However, the deep learning methods require large datasets with at least 1000–1500 data rows or wireless metrics scans. However, for our tests, we achieved good results with the CNN due to its ability of deep feature extraction. In the IPDSC Lab, which contained only two Wi-Fi networks, the CNN model performed even better than the random forest model and this confirms our thoughts. Furthermore, the models were robust to the close halls LH1 and LH2 as the detection models indicated most of the readings in their right geofences. On the other hand, Lecture Hall 26 showed the lowest accuracy using all the models due to the weak Wi-Fi coverage. The building’s accuracy using MF-ML in Figure 19 has nearly the same conclusions as the discussed indoor layer with lower accuracy around 90% average. Table 1, Table 2, Table 3 and Table 4 present more detailed results for the developed models in two indoor locations and two buildings with WF-ML and MF-ML models, respectively.

**Figure 20:**
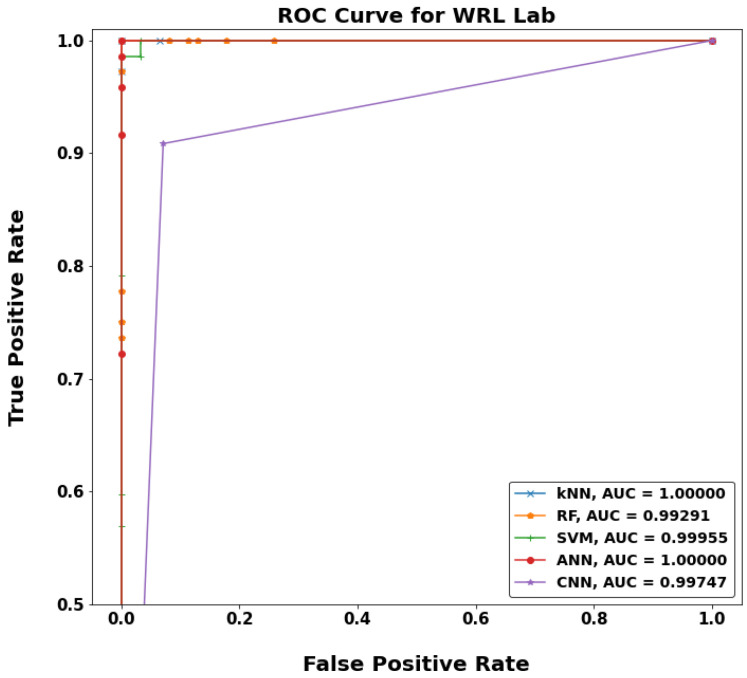
ROC curves for WF-ML models in WRL Lab.

**Figure 21:**
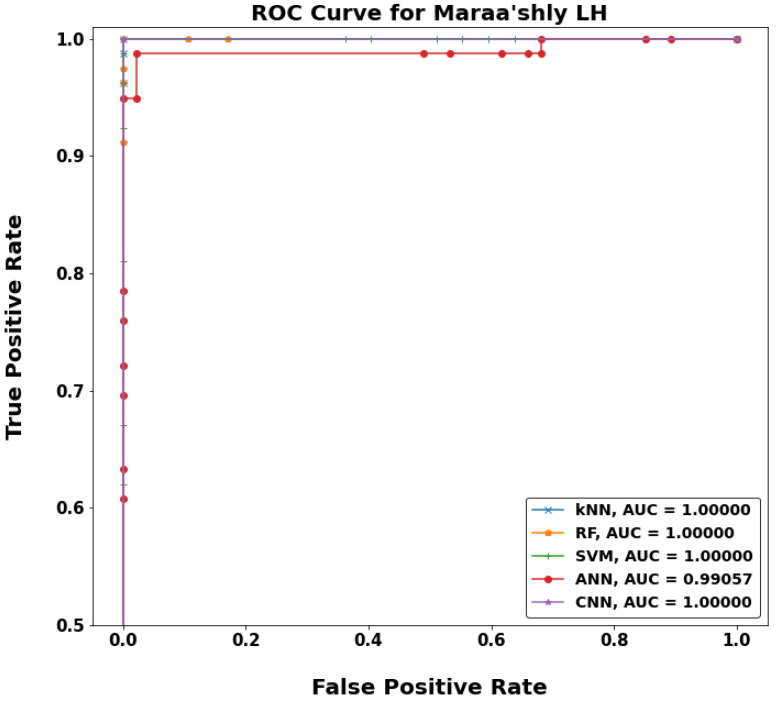
ROC curves for WF-ML models in Maraa’shly LH.

**Figure 22:**
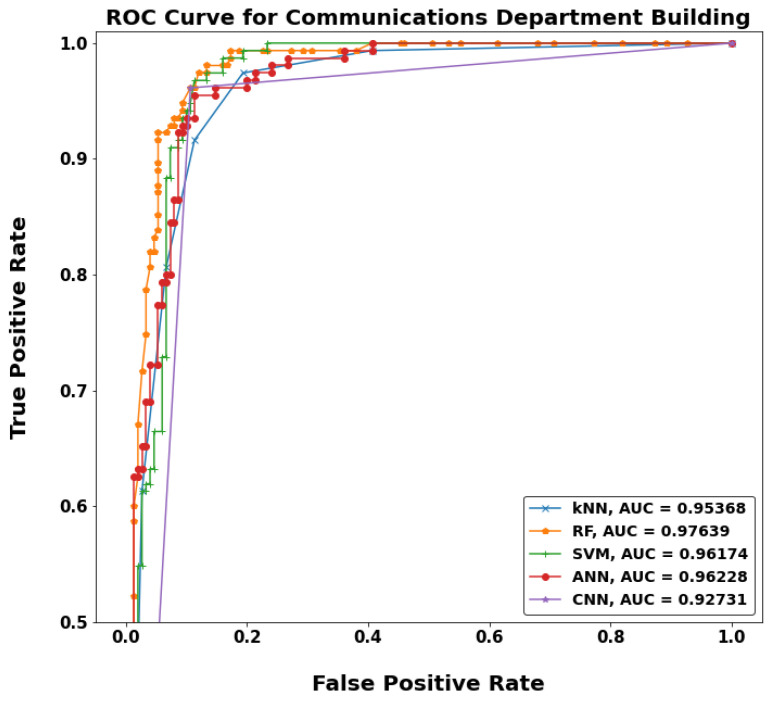
ROC curves for MF-ML models in Communication building.

**Figure 23:**
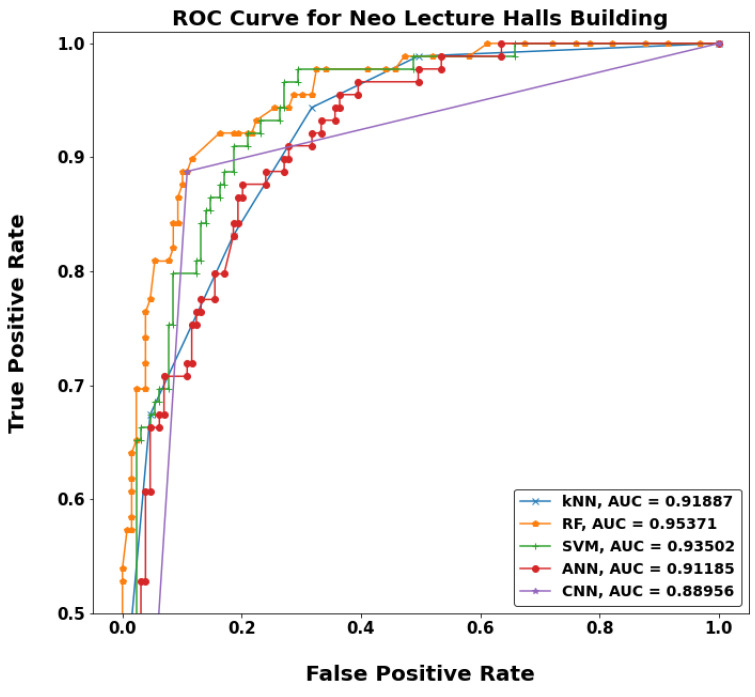
ROC curves for MF-ML models in Neo LH building.

Figure 20, Figure 21, Figure 22 and Figure 23 show a typical receiver operating characteristic (ROC) curve for the chosen models in some test locations. ROC can be considered as an evaluation metric for binary classification problems that shows the performance of a classification model at all classification thresholds. The higher the area under the curve (AUC), the better the performance of the model at distinguishing between the positive and negative classes. When AUC = 1, then the classifier can perfectly distinguish between all the positive and the negative class points correctly. If the AUC was 0, then the classifier would be predicting all negatives as positives, and all positives as negatives. When 0.5 < AUC < 1, there is a high chance that the classifier will be able to distinguish the positive class values from the negative class values. This is because the classifier can detect more numbers of true positives and true negatives than false negatives and false positives. Therefore, a higher AUC value for a classifier has the better ability to distinguish between positive and negative classes. When AUC = 0.5, the classifier is not able to distinguish between positive and negative class points, meaning either the classifier is predicting random class or constant class for all the data points.

**Figure 24:**
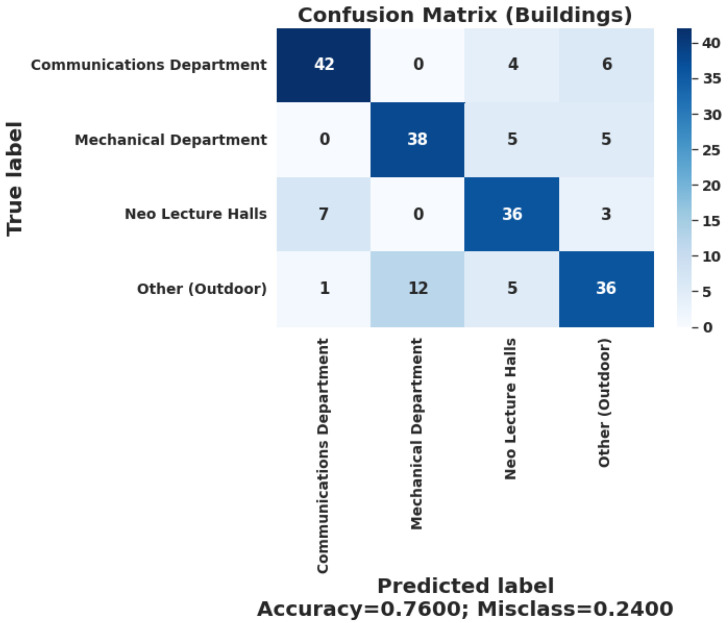
Confusion matrix of the overall system performance with MF-ML models.

**Figure 25:**
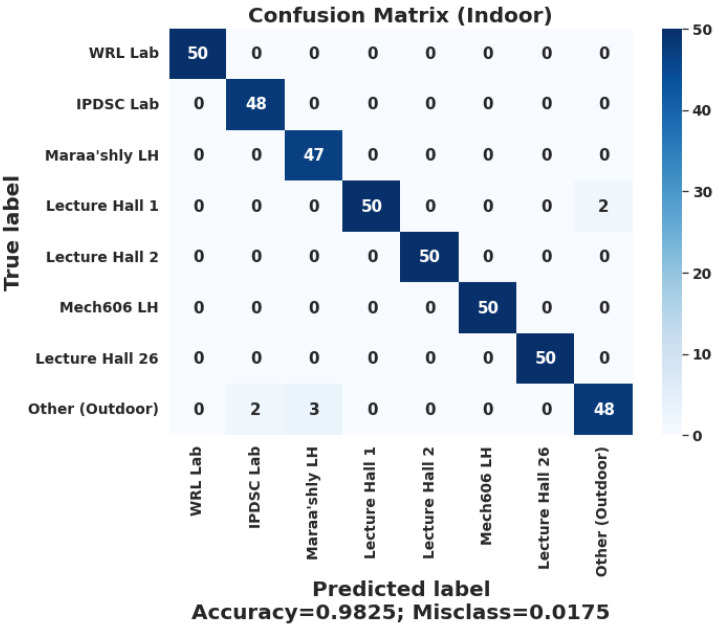
Confusion matrix of the overall system performance with WF-ML models.

Figure 24 and Figure 25 show a confusion matrix that summarizes the prediction results of the whole system when it classifies the user’s predicted geofence across all their associated geofences in the building layer and the indoor layer. The chosen ML deployed model was the ANN one due to the TensorFlow lite [38] support on the android platform in general and the lower complexity and size comparing to the CNN lite mode. Furthermore, the classical models are not supported yet by TensorFlow lite. Most of the selected algorithms’ accuracies fluctuate within a range of 2% to 4%, which is not that large in the overall prediction performance of the deployed model on the mobile application. The problems were with the inference time and the optimization of the mobile application. During testing, the ANN and CNN models were easily tested with the TensorFlow lite library, which is supported by the Flutter framework [39] that was used in the mobile application development. Flutter is an open-source UI software development kit created by Google. It is used to develop cross-platform applications for Android, iOS, Linux, macOS, and Windows. Therefore, the developed application can be deployed to work on an iOS device with no problems with the current implementation.

The inference time was nearly 0.0016 sec, with all of the ANN models and CNN models achieving 0.12 sec. However, the classical models were not supported by the TensorFlow lite library. The solution was to use another library called ml_algo [40] to test the models. The inference time of kNN was 10.18 sec as an example of the classical ML models. This number was expected as the TensorFlow lite library leverages specialist mobile accelerators and it provides on-device machine learning inference with low latency and a small binary size by decreasing the precision of the ML models. Therefore, the choice was to use the ANN model as the difference in accuracy is pretty low and it has the best inference time so far among the other algorithms.

Each geofence has taken as input 50 scans of the Wi-Fi and MF metrics to indicate the performance of the geofences classification. The buildings layer accuracy of the system is 76%. Meanwhile, the indoor layer accuracy of the system is 98.25%, which is highly acceptable taking into consideration that each location offers a significant challenge to the ML models.

### 5.3. LAGD Architecture

**Figure 26:**
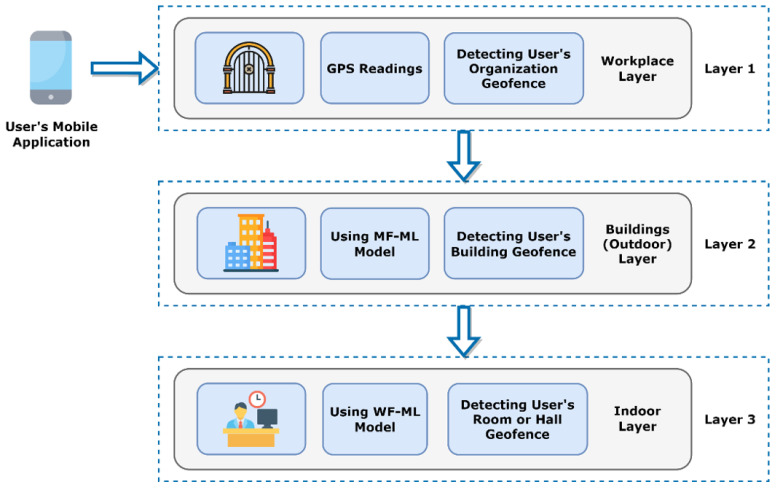
LAGD architecture.

From the results discussed along with the practical testing, the idea of the Layered-Architecture Geofence Division (LAGD) shown in Figure 26 can be introduced. The problem of using the Wi-Fi metrics only for the GDS generation is their absence in the outdoor environment. However, they provide good indication features in indoor environments. The opposite happens with the magnetic data, as they are significantly good for classifying the outdoor open area environments of the buildings from their insides. However, in indoor environments, the general readings of magnetic data are similar. The proposed solution was using the idea of active geofencing (GPS-based service) as a first layer of detecting the user’s current workplace or organization. Then, the magnetic data are used as classification features for the detection of buildings as the second layer of detection of the user by using MF-ML models. Then, after the detection of the user’s building location, the indoor rooms WF-ML models of this building are invoked for applying the third layer of detection.

The benefit of applying this technique is reducing the inference and processing time as we would invoke the geofences models only after entering the workplace. So, after the user is identified in a geofence or a hall by the WF-ML model, the LAGD architecture would only invoke the WF-ML models in the user’s detected workplace and building. Then, it would only return to the previous layers if they were not in any of the geofences of the formally detected building to recheck if the user was still in the building and the workplace. Thus, the GPS would not be used continuously but invoked only when needed to reduce power consumption and save battery life. Furthermore, taking two types of features together would require more processing power of the user’s smartphone along with increasing the power consumption. In addition, it would simplify the GDS datasets collection and the ML models creation for each type of geofence.

## 6. Conclusions

In this paper, an efficient approach is proposed to manage the COVID-19 control measures in workplaces. In this approach, the workplace environment is divided into a set of geofences. Then, the geofences are grouped to form a cluster of associated geofences for each employee using a dynamic user-centric clustering scheme based on the nature of the work of employees. For each geofence, Wi-Fi BSSID, Wi-Fi RSSI, and the spatial features for the magnetic field are used to generate a unique GDS using different ML techniques. Then, these techniques are performed a binary classification task to check whether the employee is inside or outside their associated geofences. The LAGD method is considered to reduce the inference and processing time as well as the processing overhead at the employee’s smartphone, where the first layer detects the current workplace using active geofencing, the second layer detects the building geofences utilizing MF-ML models and then the third layer distinguishes the indoor geofences using WF-ML models. Experimental results are established in a real workplace environment with the ANN deployed model and the achieved accuracy of the proposed approach is about 98.25% in indoor geofences and 76% in building geofences. The proposed approach can also be used to improve workplace operations and management by producing new features such as space management, energy management, smart attendance systems, and policy enforcement. This workplace management improvement can be done by providing useful information about the occupants’ density in different zones and their movement patterns.

## Figures and Tables

**Table 1:** WRL Lab Indoor WF-ML results.

Geofence	TSMF Data	ML Model	Accuracy	Precision	Recall	Kappa	F1	AUC
WRL Lab	WF-ML	kNN	1.0	1.0	1.0	1.0	1.0	1.0
		RF	0.99	1.0	0.98	0.98	0.99	1.0
		SVM	0.99	1.0	0.98	0.98	0.99	0.99
		ANN	1.0	1.0	1.0	1.0	1.0	1.0
		CNN	0.97	0.94	1.00	0.93	0.97	0.96

**Table 2:** Maraa’shly LH Indoor WF-ML results.

Geofence	TSMF Data	ML Model	Accuracy	Precision	Recall	Kappa	F1	AUC
Maraa’shly LH	WF-ML	kNN	0.99	1.0	0.98	0.98	0.99	1.0
		RF	1.0	1.0	1.0	1.0	1.0	1.0
		SVM	0.99	0.98	1.0	0.98	0.99	1.0
		ANN	0.97	0.97	0.98	0.94	0.98	0.99
		CNN	1.0	1.0	1.00	1.0	1.0	1.0

**Table 3:** Communications department building outdoor MF-ML results.

Geofence	TSMF Data	ML Model	Accuracy	Precision	Recall	Kappa	F1	AUC
Communications Building	MF-ML	kNN	0.90	0.89	0.91	0.80	0.90	0.95
		RF	0.93	0.94	0.92	0.86	0.93	0.97
		SVM	0.92	0.89	0.96	0.85	0.93	0.96
		ANN	0.91	0.91	0.90	0.82	0.91	0.96
		CNN	0.92	0.90	0.96	0.85	0.93	0.92

**Table 4:** Neo LH building outdoor MF-ML results.

Geofence	TSMF Data	ML Model	Accuracy	Precision	Recall	Kappa	F1	AUC
Neo LH Building	MF-ML	kNN	0.63	0.68	0.45	0.26	0.54	0.73
		RF	0.75	0.84	0.60	0.50	0.70	0.83
		SVM	0.64	0.88	0.30	0.45	0.27	0.73
		ANN	0.70	0.86	0.47	0.40	0.60	0.73
		CNN	0.88	0.84	0.88	0.77	0.86	0.88

## Data Availability

The data presented in this study are available on request from the corresponding author.
